# Polycystic Ovary Syndrome and Endocrine Disruptors (Bisphenols, Parabens, and Triclosan)—A Systematic Review

**DOI:** 10.3390/life13010138

**Published:** 2023-01-04

**Authors:** Tinkara Srnovršnik, Irma Virant-Klun, Bojana Pinter

**Affiliations:** 1 Division for Women’s Healthcare, Šiška Unit, Community Health Centre Ljubljana, Metelkova ulica 9, 1000 Ljubljana, Slovenia; 2 Faculty of Medicine, University of Ljubljana, Vrazov Trg 2, 1000 Ljubljana, Slovenia; 3 Clinical Research Centre, University Medical Centre Ljubljana, Vrazov Trg 1, 1000 Ljubljana, Slovenia; 4 Division of Obstetrics and Gynecology, University Medical Centre Ljubljana, Šlajmerjeva 3, 1000 Ljubljana, Slovenia

**Keywords:** endocrine disrupting chemicals, PCOS, BPA, ovarian reserve, androgens, oxidative stress

## Abstract

Exposure to endocrine disrupting chemicals (EDCs) can result in alterations of the female reproductive system, including polycystic ovary syndrome (PCOS). The aim of this review was to summarize the knowledge about the association of EDCs (bisphenols, parabens, and triclosan) with PCOS. We conducted an electronic literature search using PubMed for studies published between January 2007 and October 2022 on EDCs related to PCOS, and evaluated the association of PCOS with bisphenols, parabens and triclosan in 15 articles. Most studies revealed significantly higher plasma, urinary or follicular fluid levels of bisphenol A (BPA) in women with PCOS, and some showed a positive correlation of BPA with insulin resistance, polycystic morphology on ultrasound, hepatic steatosis, bilirubin levels, as well as free androgen index, androstenedione and testosterone serum levels, and markers of low-grade chronic inflammation. There was a negative correlation of BPA with markers of ovarian reserve, sex hormone binding globulin and vitamin D–binding protein. Parabens and triclosan have been studied in only one study each, with no significant associations with PCOS observed. Our review revealed an association of BPA with PCOS and negative effects of BPA on human ovaries; more research is needed to assess the potential associations of parabens and triclosan with PCOS.

## 1. Introduction

Increased global industrialization has exposed humans to variety of industrial chemicals which can interfere with the endocrine system [1]. As such, they have been defined as »endocrine disruptors« [2], also known as endocrine disrupting chemicals (EDCs). Several definitions of EDCs exist. The definition of the International Programme on Chemical Safety that defines the endocrine disruptor as an exogenous substance or mixture that alters function(s) of the endocrine system and consequently causes adverse health effects in an intact organism, or its progeny, or (sub)populations, is used frequently [3]. EDCs are found abundantly in the environment, resulting in daily individual exposure and accumulation over the long term. Human exposure to EDCs occurs most frequently by ingestion of contaminated food and to some extent by inhalation of polluted air and dermal uptake [4]. A wide variety of chemical compounds, both natural and synthetic, have been recognized to possess endocrine disrupting activity, including phytoestrogens, compounds in pharmaceutical and cosmetic products (e.g., parabens, triclosan (TCS)), pesticides, plastics (bisphenol A (BPA) and its alternatives), plasticizers (phthalates), metals (heavy metals, trace elements) and industrial chemicals with by-products [4,5,6].

Currently, it is established that EDCs may act via genomic pathway by binding to nuclear hormone receptors (e.g., estrogen receptors (ER), androgen receptors (AR) or progesterone receptors (PR)) exerting agonistic or antagonistic effects, or via non-genomic pathways by binding to membrane ER, PR or G-protein-coupled proteins, but some studies also revealed other possible modes of actions, such as oxidative stress, genetic susceptibility and epigenetic modifications (e.g., DNA methylation) [5,7,8,9,10]. By interfering with receptor binding, steroidogenesis and metabolism of hormones, EDCs have been shown to result in morphological and functional alterations of the female reproductive system which can result in infertility, irregular menstrual cycles, endometriosis, uterine fibroids, precocious puberty or premature ovarian insufficiency (POI), gynaecological cancers and polycystic ovary syndrome (PCOS) [1,5,11,12,13,14].

PCOS is the most common, heterogenous and multifactorial endocrine disorder, affecting 5–10% women of reproductive age [15]. According to a consensus meeting sponsored by the European Society of Human Reproduction and Embryology/American Society for Reproductive Medicine (ESHRE/ASRM), PCOS can be diagnosed if two out of the following three criteria are fulfilled: (i) clinical and/or biochemical hyperandrogenism, (ii) chronic oligomenorrhea and/or anovulation or (iii) the presence of polycystic ovaries on transvaginal ultrasonography [16]. The pathophysiology of this endocrinopathy still remains unclear. However, recently it has become increasingly apparent that genetic, epigenetic, endocrine, metabolic as well environmental factors may all contribute to the development of this disorder [14,17,18]. Regarding hormonal and metabolic abnormalities of women diagnosed with PCOS, EDCs in the environment may be particularly relevant to consider [17,19]. EDCs, bisphenols, parabens and triclosan are found in several daily used products and are present as high production volume compounds manufactured worldwide. Therefore, the aim of our systematic review was to review the current knowledge on possible associations of bisphenols, parabens and triclosan with PCOS.

## 2. Materials and Methods

### 2.1. Literature Identification

This systematic review is reported in accordance with the Preferred Reporting Items for Systematic Reviews and Meta-analyses (PRISMA) guideline. We conducted an electronic literature search utilizing the National Library of Medicine (PubMed) database. The following medical subject heading (MESH) keywords were used to search for the studies reporting on the role of environmental endocrine disruptors in the occurrence of PCOS in women: »polycystic ovary syndrome« OR »PCOS« AND »endocrine disruptor«. The database was searched for studies published from January 2007 until 1 October 2022.

### 2.2. The Inclusion and Exclusion Criteria

Studies included were randomized control studies, case-control studies, cross-sectional studies, pilot studies or cohort studies in human. Studies conducted other than in humans, reviews, and studies published in languages other than English, were excluded from this review. We also performed a hand search of the reference lists of full-text articles that met our criteria in the primary literature search. All manuscripts and abstracts were independently reviewed by all three investigators for possible study inclusion regarding their subject and quality. The selected articles were read in full to confirm eligibility and to extract data. Disagreements were resolved by scientific discussion, if necessary.

### 2.3. Data Extraction

From the included studies, the following information was extracted for detailed evaluation: characteristics of the included studies (first author, year of publication and country), study design characteristics (study design, sample type, methods including statistics), sample size, type of endocrine disruptor and main findings.

## 3. Results

For this review, we included studies identifying possible association of exposure to bisphenols, parabens and triclosan with PCOS.

### 3.1. Bisphenol A (BPA)

BPA (2,2-bis (4-hydroxyphenol) propane) is one of the most abundant chemicals produced worldwide and one of the most extensively studied EDCs. It is used for production of polycarbonate plastics used in numerous common products (e.g., optical, media, electrical and electronics, houseware and appliances, medical devices, dental materials, reusable and baby plastic bottles), as an essential component of epoxy resins that are mainly used to coat the inner surface of food and beverage metallic cans, and as antioxidant or inhibitor of polymerization in some plasticizers, polyvinyl chloride, paper, paper products and thermal receipt papers. BPA is also detected in dust, air, drinking water, surface water and wastewater. The main human exposure to BPA occurs due to ingestion of contaminated food and beverages from plastic food packaging and bottles, although its abundant distribution in the environment leads also to contamination via the skin, or via inhalation of household dusts [18,20,21,22,23,24]. BPA is detectable in various body fluids including urine, serum, saliva, follicular fluid, breast milk, colostrum, placenta, umbilical cord and amniotic fluid [25,26,27]. It is glucuronidated by liver microsomes and catalysed by an isoform of uridine diphosphate-glucuronosyl transferase (UGT) enzyme, and then rapidly excreted in the feces and urine [28].

BPA exerts weak antiandrogenic, estrogenic and antithyroid activities, its estrogenic activity being 1000–100,000-fold less than that of estradiol. By binding to the AR, BPA acts as its antagonist when its level is high [29]. In addition, there is some evidence that BPA affects epigenetic mechanisms such as DNA methylation and chromatin structure [30]. Some studies have revealed that elevated serum or urinary BPA levels were associated with anovulation, decreased number of antral follicle count (AFC) or lower level of anti-Müllerian hormone (AMH), and consequently infertility [26,27,31]. Due to the constant exposure, BPA possesses a real hazard to human health and has thus been banned in baby bottles in the European Union in 2011; the tolerable daily intake of BPA is considered 0.05 mg/kg bodyweight per day [32]. Such regulations have led to the development of presumably less harmful BPA analogues such as bisphenol S (BPS) and bisphenol F (BPF). However, due to structural similarities with BPA, these alternatives are also hormonally active by causing estrogenic, antiestrogenic, androgenic and antiandrogenic effects [21,33,34]. More research is needed in assessing the reproductive health risk of BPA analogues.

The potential role of BPA in the pathogenesis of PCOS is still not well understood. Although there are many reports on the effects of BPA on the ovarian steroidogenesis, for example an interaction with transmembrane ERs such as G protein coupled receptor 30 or inhibition of aromatase activity, mechanisms of actions remain unclear [35]. Animal models in adult rats have shown potential upregulation of the gonadotropin-releasing hormone (GnRH) pulse generator activity in adult life when exposed to BPA in neonatal period [36]. Changes of the hypothalamic pituitary-ovarian axis have also been reported with interruption of gonadotrophin secretion or as interruption of hypothalamic GnRH release [37]. BPA administration has been associated with modifications of ovarian steroidogenic enzymes including 17β hydroxylase, cholesterol side chain cleavage enzyme and steroidogenic acute regulatory protein, some of which are also implicated in PCOS hyperandrogenism [37]. BPA has also been shown to directly stimulate androgen synthesis in the ovarian theca-interstitial cells [38] and decrease the estradiol production in the granulosa cells [39,40]. In addition, BPA has been shown to displace sex steroids from sex hormone binding globulin (SHGB), therefore increasing the amount of free testosterone [41]. As PCOS is considered as a proinflammatory state, chronic low-grade inflammation also seems to emphasize the ovarian dysfunction and the exaggerated theca cells androgen production [42]. A considerable number of in vitro and in vivo animal studies in rodents and mammalians indicate that developmental BPA exposure disrupts oogenesis and early oocyte meiosis as well as normal follicle growth resulting in accelerated follicle transition and increased incidence of atretic follicles [37].

Hyperinsulinemia and obesity are common features in women with PCOS. The results obtained from the studies in vitro and in vivo revealed a great potency of BPA for non-genomic activation of adipogenic transcription factors [43], up-regulation of adipogenic genes [44] and lipid accumulation [45]. In human adipose tissue, BPA stimulates the release of cytokines favouring adiposity namely interleukin-6 (IL-6) and tumor necrosis factor alpha (TNF-α), whereas it inhibits the release of adiponectin that acts protective against metabolic syndrome [37]. By acting via ERs on adipocytes and macrophages in the adipose tissue, BPA promotes inflammatory conditions [35]. BPA might as well be involved in the alteration of glucose homeostasis [46] and in the development of hyperinsulinemia and type 2 diabetes mellitus [47,48]. Moreover, animal models have shown that BPA can increase insulin secretion [49]. As the possible missing link between insulin resistance and PCOS, vitamin D deficiency has also been proposed [50]. BPA has been shown to influence the epigenetic mechanisms by directly inducing epigenetic changes in the genome [51] or by hyperandrogenemia which influences DNA methylation [52].

### 3.2. Parabens

Parabens are a group of alkyl esters of p-hydroxybenzoic acid that are used as antimicrobial agents and preservatives in personal care products (PCP), foods and pharmaceuticals. They are also found in indoor dust. Methylparaben (MP) and propylparaben (PP), accompanied by ethylparaben (EP), butylparaben (BP) and benzylparaben (benzylP) are among the most used. The antimicrobial activity of parabens is related to the length of the alkyl chain of the individual esters. The activity of parabens increases along with increasing side-chain length. The main route of parabens exposure is considered to be dermal absorption from personal care products, but exposure can occur also through ingestion and inhalation [11,53,54,55]. Parabens, and predominantly their conjugates, can be measured in urine and have been shown to be valid biomarkers of exposure [56]. However, they have been extracted also from breast milk, colostrum, blood and seminal plasma [57].

Parabens exert estrogenic effects through ER that increase with the length and branching of the alkyl chain [55,58]. An increase in the size of the alkyl group may intensify paraben transactivation of ERs in vitro [59]. In addition, parabens can mimic the effects of 17β-estradiol by binding to ERs and a linear relationship between parabens and cell proliferative potency, as well as ER-binding potency, has been reported [59]. In animal models, parabens were also shown to act as antiandrogens and affecting thyroid function [59,60]. Data on the possible modes of action and female reproductive health effects of paraben exposure are limited. In one of the animal toxicity studies, parabens decreased ovarian weight and caused histopathological changes in the ovaries such as a decrease in corpora lutea, an increase in number of cystic follicles and thinning of follicular cells as well as the decrease in serum estradiol levels [59]. On the contrary, others reported that the effects of parabens on reproductive organ weights and histopathological abnormalities in female offspring have not been observed [61]. Since many scientific reports suggest that everyday use of cosmetics containing parabens can exert harmful effects on human body, parabens have as well been—similarly to BPA—the subject of regulation by regulatory organs; in the European Union it is recommended that BP and PP should be prohibited in leave-on-the-skin cosmetic products intended for use on the nappy area of children aged [55].

Studies investigating the potential associations between parabens and female reproductive health are sparse. Higher urinary levels of parabens were associated with reduced antral follicle count [62,63], a shorter length of the menstrual cycle [64] and decreased odds of becoming pregnant [65]. Paraben levels measured during pregnancy were associated with a decreased ratio of estradiol/progesterone and increased levels of SHBG, indicating that parabens may decrease estradiol availability during pregnancy [66]. However, others found no association between urinary concentrations of parabens with in vitro fertilization outcomes [67,68].

### 3.3. Triclosan (TCS)

Triclosan (2, 4, 4 1-trichloro-2 1-hydroxy-diphenyl ether) is widely used in personal care, household, pharmaceutical, veterinary and industrial products due to its broad-spectrum antibacterial and antifungal properties. TCS usually enters the body by ingestion or dermal and mucosal contact, although inhalation of products including spray deodorants or air fresheners may also be the route of exposure. It can be detected in human urine, blood, breast milk and amniotic fluid [11,69].

TCS can bind to ER and has a higher binding affinity to ERα over ERβ; in several experimental modalities TCS amplified the estrogenic effects of ethynil estradiol [70]. Evidence suggests a variety of hormonal activities of TCS, including estrogenic, antiestrogenic and antiandrogenic activities all of which can be related to PCOS-like syndrome [60,70]. However, data on association of TCS with thyroid function in pregnant women remain conflicting [66,71]. In the European Union, it is not appropriate to approve TCS for use in human hygiene biocidal products [72].

In zebrafish, TCS has been linked to oxidative damage in the ovaries and advance reactive oxygen species-dependent ovary apoptosis by down-regulating the expression of antioxidant-related genes [73]. Furthermore, exposure to TCS could result in decreased levels of gonadotrophins, progesterone and GnRH, as well as a reduction in antral follicles and corpora lutea [74]. In cultured human ovarian granulosa cells, TCS disrupted mitochondria membrane potential and exhibited mitochondria toxicity, increased estradiol and progesterone levels and promoted toxicological effects on steroidogenesis. In mammalian cells, TCS impaired mitochondrial oxidative phosphorylation [75].

In human studies, TCS exposure may negatively affect AFC [76,77]. An association between female fertility and urinary concentration of TCS remains uncertain and conflicting [65,76,78,79].

**Figure 1:**
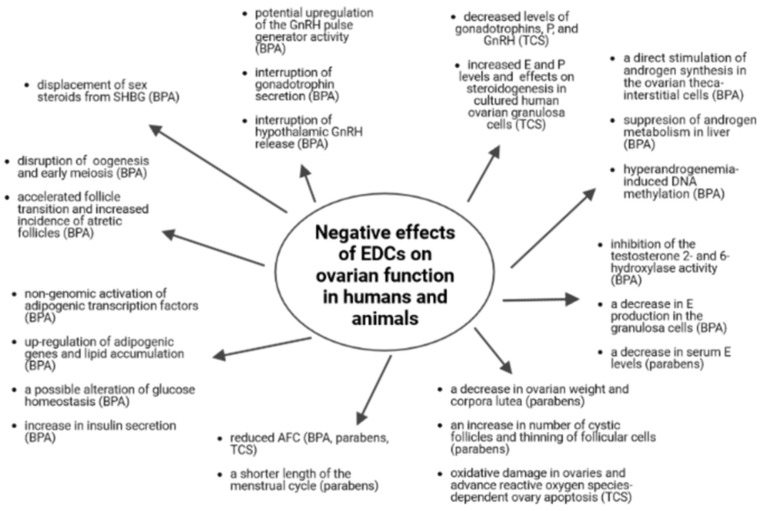
The possible effects of endocrine disrupting chemicals—bisphenols, parabens and triclosan—on ovarian function in humans and animals. Abbreviations: BPA, bisphenol A; E, estradiol; EDCs, endocrine disrupting chemicals; GnRH, gonadotropin-releasing hormone; P, progesterone; SHBG, sex hormone binding globulin; TCS, triclosan.

The possible effects of endocrine disrupting chemicals—bisphenols, parabens and triclosan—on ovarian function in humans and animals are summarized in Figure 1.

### 3.4. Literature Search

**Figure 2:**
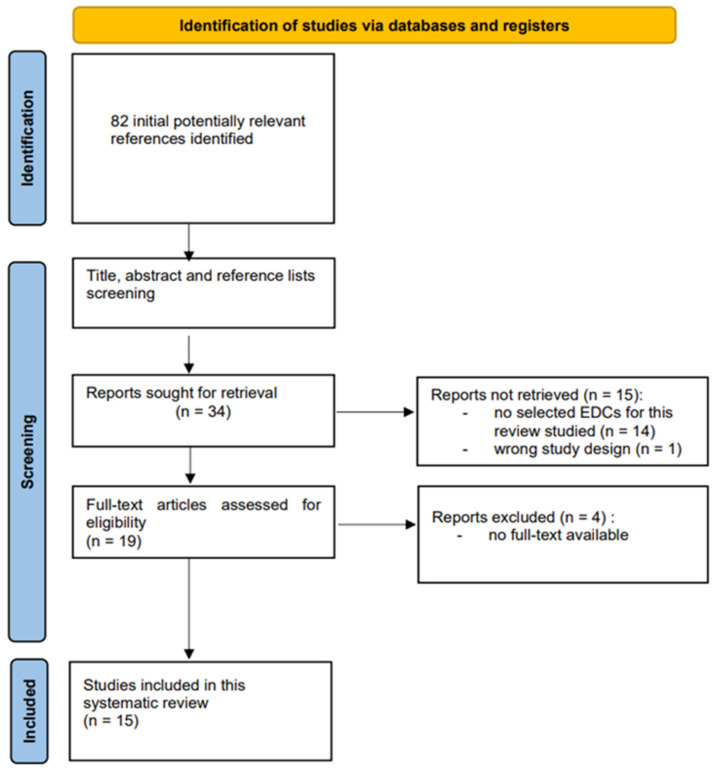
PRISMA flow diagram of the study selection process. Association of endocrine disrupting chemicals—bisphenols, parabens and triclosan—with polycystic ovary syndrome.

After the primary literature search, we identified 82 potentially eligible citations. Based on our inclusion criteria, we selected 16 studies for initial screening. By reviewing the reference lists of selected studies, we revealed an additional 89 potentially eligible citations. After removing duplicates, review articles and studies conducted other than in humans, 19 suitable studies met our inclusion criteria. Only studies identifying possible association of exposure to bisphenols, parabens and triclosan with PCOS were included in this review. After eliminating references with no full text available [80,81,82,83], finally 15 studies were included for this review (Table 1). Figure 2 presents a PRISMA flow diagram of the association of endocrine disrupting chemicals—bisphenols, parabens and triclosan—with PCOS.

### 3.5. Association of Endocrine-Disrupting Chemicals with Polycystic Ovary Syndrome

In Table 1 the results of the systematic review on association of endocrine-disrupting chemicals—bisphenols, parabens and triclosan—with PCOS are presented. Statistically significant differences are presented.

#### 3.5.1. Bisphenol A and PCOS

In this literature review, significantly increased serum, urinary or follicular fluid BPA levels in PCOS population were found in the majority (11) of studies. Comparison of BPA levels between PCOS and control women showed significantly higher urinary (3.34 ± 2.63 vs. 1.43 ± 1.57 ng/mL) [87], blood (1.05 ± 0.56 vs. 0.72 ± 0.37 ng/mL) [89] and follicular fluid (440.50 ± 63.70 vs. 338.00 ± 57.88 pg/mL) [97] levels of BPA in PCOS women than in controls with generally higher concentrations of BPA in urine and blood than in follicular fluid. However, this has not been found in some other studies [86,88,95,98]. In addition, in PCOS population clearance of BPA was associated with certain UGT polymorphisms; the secretion of androgens was associated with UGT polymorphisms along with the BPA level in serum [92]. There was a positive correlation between urinary BPA levels and polycystic morphology of ovaries observed on ultrasound [84]. Thirteen studies focused on the hormonal status in PCOS women with regards to BPA exposure, some of them expanding the research to metabolic and pro-inflammatory parameters associated with PCOS [84,85,86,88,89,90,91,92,93,94,96,97,98]. In the study by Kandaraki et al., there was a positive correlation of BPA with testosterone (T) and androstenedione (A) in the total group [89], while a study by Konieczna et al. revealed a positive correlation of BPA with the total T and free androgen index (FAI) only in PCOS group [90]. Akin et al. observed a positive correlation of serum BPA levels with total T, free T, dehydroepiandrosterone sulfate (DHEAS) and Ferriman–Gallwey score in the entire study group. In PCOS group, there was a positive correlation of BPA with total T and DHEAS levels [85]. In a study by Šimkova et al., higher levels of BPA, luteinizing hormone (LH), luteinizing hormone/follicle–stimulating hormone ratio (LH/FSH ratio), T, free T, bioavailable T, A and 7β-OH-epiandrosterone were found in normal-weight PCOS women. There was a positive correlation of BPA with T, SHBG, FSH and LH in healthy controls [93]. On the other hand, a study by Lazurova et al. showed a significant negative correlation of urinary BPA levels with steroid hormones levels in PCOS women, such as estradiol (E), total T, free T as well as FAI [91].

Regarding the vitamin D deficiency in PCOS women in relation to VDBP and BPA, we found only one study assessing VDBP concentrations in PCOS women. As serum BPA, VDBP, 25(OH)D levels and the frequency of vitamin D deficiency were comparable between PCOS and control group, PCOS group women showed a negative correlation between their serum BPA and VDBP levels as well as SHBG levels. There was a positive correlation of serum BPA with bilirubin levels in the PCOS group, control group and the whole study group of women [88].

There were ten studies which were studying metabolic abnormalities in PCOS women with regards to BPA levels in their biological samples [84,85,88,89,90,91,93,94,95,96]. Lazurova et al. demonstrated that PCOS women with high-BPA levels had significantly higher serum insulin and Homeostatic Model Assessment for Insulin Resistance (HOMA IR) compared to low-BPA PCOS women, with no significant difference in body mass index (BMI) among both groups [91], similarly to Kandaraki et al. and Tarantino et al. [89,94]. There are inconsistencies in results of studies evaluating relationship between BMI and BPA in PCOS women. Some studies found a positive correlation between BPA and BMI [87,89], while others did not confirm such relationship [84,90].

Markers of low-grade inflammation in PCOS women in relation to BPA have been studied in two studies included in our review [93,94]. The study of Tarantino at al. revealed higher serum BPA levels to be associated with higher levels of low-grade chronic inflammation markers [94].

In our literature search, we found three studies which observed possible association of BPA with markers of ovarian reserve in PCOS women [93,97,98]. One study showed a significant inverse association between urinary BPA concentration and AFC in women with PCOS [98].

In two studies of this literature review, the authors failed to prove an association between BPA levels and PCOS in women [86,95].

#### 3.5.2. Parabens and PCOS

In our systematic literature review, there was only one study which studied the potential association of parabens with PCOS in women. The exposure to selected parabens was comparably low among all studied groups of women and no differences were observed in the plasma paraben concentrations in relation to PCOS [93].

#### 3.5.3. Triclosan and PCOS

We found only one study exploring the possible association between triclosan and the risk of PCOS in women, with no significant associations found between urinary TCS concentrations and PCOS observed [86].

## 4. Discussion

BPA is a ubiquitous contaminant with a broad spectrum of applications. It is the most common environmental pollutant incriminated for reproductive and metabolic disorders resembling PCOS in animal models. Therefore, most published literature concerning EDCs and PCOS is limited mostly to BPA. In our literature review, significantly increased serum or urinary BPA levels in PCOS population compared to healthy controls were found in majority of studies performed in humans [84,85,87,89,90,91,92,93,94,96,97]. The results of included studies suggested that BPA is associated with PCOS in women and may negatively affect the ovaries in various ways.

The studies included in our review showed an association between BPA concentrations and androgens in PCOS women. The first observation of increased serum BPA levels in PCOS women was reported by Takeuchi et al. in 2004. This study revealed significant positive correlations between serum BPA levels and total T, free T, A and DHEAS, suggesting a strong relationship between serum BPA and androgen concentrations, speculatively because of androgens on the metabolism of BPA. As it has already been reported that the level of UGT activity is downregulated by androgens, the glucuronidation of BPA might be suppressed under the hyperandrogenic environment, observed in PCOS women. Possible explanations for these findings could be: (i) BPA-stimulated androgen production or (ii) differences in BPA intake, metabolism and excretion [99]. Our review showed that clearance of BPA and androgens was associated with certain UGT polymorphisms in PCOS population [92]. Moreover, BPA has been shown to directly stimulate androgen synthesis in the ovarian theca-interstitial cells [38] and inhibit the activity of two different testosterone hydroxylases (2- and 6-hydroxylase), leading to decreased testosterone catabolism and indirectly to increased testosterone concentrations [100]. Similarly, thirteen studies included in our review focused on the hormonal status in PCOS women regarding BPA exposure, confirming positive correlation of BPA with androgens in PCOS group [85,90,93] or total group of women [89].

Although more investigators describe a positive correlation of BPA with androgens either through stimulation of androgen synthesis or suppression of androgen metabolism in the liver, some other authors hypothesize that elevated BPA probably represents a consequence but not a cause of PCOS, as elevated androgens may decrease BPA clearance [36]. BPA could also have a possible suppressive effect on ovarian steroidogenesis, thus further confirming the controversial relationship between BPA and ovarian steroids [101].

The study by Akgul et al. established positive correlation of urinary BPA levels with polycystic morphology on ultrasound in adolescents aged 12–18 years [84]. Altered ovarian morphology in association with BPA is also in line with findings of Fernandez et al. in rats where they described a large number of ovarian cysts in rats that had been exposed to high doses of BPA [36]. Well-known neuroendocrine dysfunction in PCOS—acceleration of the GnRH pulse generator activity and thus inappropriate gonadotropin synthesis and release, with elevated LH and low FSH level—leads to increased androgen production from theca cells and impaired compensatory aromatization to estrogens in the granulosa cells and impaired follicular development [102]. Developmental exposure to BPA has shown potential upregulation of the GnRH pulse generator activity in adult life when exposed in neonatal period [36]. In addition, changes in interruption of gonadotrophin secretion or hypothalamic GnRH release have also been reported [37].

BPA is a potent SHBG ligand which displaces androgens from SHBG binding sites and increases free T [41]. SHBG is secreted by the liver along with other proteins, e.g., VDBP. Vitamin D deficiency has been shown to be highly prevalent in PCOS population and possibly contributing to metabolic disturbances in PCOS [50]. The study by Jedrzejuk et al. assessed VDBP and SHBG concentrations in PCOS women regarding BPA and showed a negative correlation between VDBP, SHBG and BPA serum levels. On the other hand, there was a positive correlation of BPA with bilirubin levels in the PCOS group of women [88]. Although not evaluating bilirubin levels, this is partially in line with results of the study by Tarantino et al. where increased BPA levels positively correlated with laboratory liver tests (AST, ALT, GGT) in PCOS group of women [94]. A study by Jedrzejuk et al. was the first to have revealed a significant relationship between serum BPA and bilirubin levels in women with PCOS postulating that bilirubin levels can be the most sensitive marker of early liver dysfunction associated with exposure to BPA, the higher AST/ALT ratio in women with PCOS thus supporting this concept [88].

The studies included in our review showed an association between BPA levels and metabolic abnormalities in PCOS women. The studies on metabolic abnormalities in PCOS women with regards to BPA by Kandaraki et al. [89] Lazurova et al. [91] and Tarantino et al. [94] demonstrated a positive correlation between BPA levels and severity of insulin resistance in women with PCOS independently of obesity, or BMI. However, meta-analysis conducted in 2017 concluded that high BPA levels showed significant association with high BMI and high HOMA-IR [19]. In a study by Šimkova et al., higher BPA levels were found in both normal-weight and obese PCOS women, but only obese PCOS women had significantly higher HOMA-IR, fatty liver index, triglycerides and significantly lower high-density lipoprotein (HDL) cholesterol [93]. This is partially in line with a study by Vahedi et al., where all participants were BMI-matched and there were no significant differences in BMI, but the BPA, serum triglyceride and cholesterol levels in PCOS group were significantly higher [96]. Tarantino et al. displayed increased prevalence in hepatic steatosis in PCOS women reflecting a possible BPA-induced oxidative damage in the liver caused by reactive oxygen species [94]. Contrarily, Konieczna et al. observed that there were no significant differences in HOMA-IR, serum total cholesterol, HDL cholesterol or triglicerides in between studied groups [90]. Obviously, there are discrepancies in results of studies evaluating relationship between BMI and BPA in PCOS. Some other studies on relatively small groups of women found a significant positive correlation between BPA and BMI [87,89,101], while others did not confirm such relationship [84,90].

Regarding markers of low-grade inflammation, there was a strong association of increased BPA levels with markers of low-grade chronic inflammation (CRP, IL-6 and spleen enlargement) in PCOS group reported in a study by Tarantino et al. The authors thus hypothesized that besides the direct hepatotoxic and adipogenic effects, BPA could act as pro-inflammatory primer and therefore spleen enlargement could represent a marker of this process, possibly linked to the immune derangement in women with PCOS [94].

There were concerns raised that environmental BPA exposure may adversely affect the oocyte quality and cause the decline of ovarian reserve and fertility in the general population. In our review, one study revealed a significant inverse association between urinary BPA concentration and AFC in women with PCOS [98], which is in line with some previous studies where higher urinary BPA concentrations were associated with diminished ovarian reserve [26,31,103]. On the contrary, Silvestris et al. found no correlation between urinary BPA levels and serum AMH concentrations [104]. In a study by Wang et al., the BPA concentration was higher in the follicular fluid from PCOS women when compared to non-PCOS women. Moreover, after in vitro BPA treatment, decreased aromatase expression and estradiol synthesis in granulosa cells were observed only in PCOS patients [97], which is in line with observations in women undergoing in vitro fertilization (IVF) treatment, where an inverse association between urinary BPA concentrations and oocytes maturation, number of oocytes retrieved and peak estradiol levels was reported [105].

Concerning BPA analogues, there has been only one study conducted in our literature search [93]. The researchers found only low plasma levels of BPS slightly more frequently in PCOS patients, but no BPF and BPAF in any sample. They concluded that if significant exposure to other alternative bisphenols in PCOS occurred, the same effect, as in the case of BPA, could be expected. On the contrary, Jurewicz et al. detected BPS levels significantly higher in PCOS women than in control subjects. Furthermore, when studying the association between serum BPA analogues and PCOS it turned out that women whose serum BPS levels were in the first tertile were more likely to be diagnosed with this endocrinopathy [34]. However, they used a single serum sample in each woman which may not have reflected the real exposure of women to BPA, BPS or BPF. Eladak et al. first reported that BPS and BPF may be detrimental to physiologic functions in both humans and rodents, addressing urgent focus on the human health risk assessment of BPA substitutes as metabolism and mechanism of action are similar to BPA [21]. In vitro studies also showed that BPS exposure may cause oxidative stress or induce obesity in women, and its endocrine activities may also be affected by its metabolites [106]. In recent years, when substitutes of BPA have been applied in numerous consumer products with high human contact, researchers conclude that the use of the bisphenol class of compounds as replacements for BPA should be implemented with caution [21,33] and needs to be further researched.

Nonetheless, it is difficult to compare the results of all these studies because of different patient selection and ethnicities, study protocols, biological material collected and methods used to assess BPA levels. In addition, due to short BPA half-life in humans and variability of the exposure over time it needs to be considered that a single urine or blood sample may not be representative of the overall exposure of an individual; a collection of 24-h urine sample or repeated measurements would be more informative. Further research is needed, preferably multicenter studies with the inclusion of a larger number of women according to the same, strict inclusion criteria and methodology.

Unlike BPA, we identified only one study studying the association of parabens with PCOS. Plasma levels of selected parabens were comparably low among all studied groups, including PCOS groups, and no differences were observed [93]. To this date, this has been the only study investigating possible association of parabens with PCOS. Only a limited number of studies explored reproductive effects of exposure to parabens on the human female reproductive health including infertility (65); preconception urinary concentrations of MP in the highest quartile and EP concentrations in the third quartile among female partners were associated with a 34% reduction in couple fecundity in partner-specific and couple-based exposure models [65]. Parabens have also been correlated with the urinary biomarker of oxidative stress, 8-hydroxy-2′-deoxyguanosine [107], revealing a possible association with PCOS.

We also found only one study exploring the possible association between TCS and the risk of PCOS [86]. In this study, no significant associations between TCS exposure and PCOS were observed. On contrary, Ye et al. found that PCOS group had significantly elevated LH/FSH ratio and higher urinary TCS levels compared to controls, with higher TCS levels associated with an increased odd of PCOS [108]. The limitations of this study were its cross-sectional design, with TCS levels measured at a single point after the onset of PCOS.

Further research is needed on the association of EDCs and PCOS in women, especially for parabens and triclosan. It is complex research, as women can be exposed to various EDCs and also other harmful environmental influences at the same time. Further research with a clear design is needed, preferably multicenter studies with the inclusion of a larger number of women. Of course, the important issue of preventive protection of women against EDCs and curatives in case of toxicity of these substances also arises.

## 5. Conclusions

The pathophysiology of PCOS still remains unclear and regarding hormonal and metabolic abnormalities of women diagnosed with PCOS, EDCs in the environment may be relevant to consider. In this systematic review, we summarized the knowledge about the association of EDCs (bisphenols, parabens and triclosan) with PCOS in the last 15 years. In most studies, our review revealed significantly higher plasma, urinary or follicular fluid levels of BPA in women with PCOS compared to healthy controls. Some studies have shown a positive correlation of BPA with insulin resistance, polycystic morphology on ultrasound, hepatic steatosis, bilirubin levels, as well as free androgen index, androstenedione and testosterone serum levels and markers of low-grade chronic inflammation. There has been a negative correlation of BPA with markers of ovarian reserve, sex hormone binding globulin and vitamin D–binding protein reported. Our findings thus confirmed an association between BPA and PCOS. Regarding parabens and triclosan, studied in only one study each, no significant associations with PCOS have been observed. According to our review, more research is needed to assess the associations of parabens and triclosan.

However, main findings of this review should be interpreted with caution due to different patient selection and small number of patients included in studies, different study protocols and biological material collected as well as different methods used to assess EDC levels. Also, humans may be exposed to many EDCs at the same time and exposure varies over time, what makes it impossible to evaluate potential synergistic or antagonistic effects of other environmental factors. As many of the EDCs studied have a very short urinary elimination half-life in human body, it is necessary to consider that a single urine or blood sample may not be representative of the overall exposure of an individual.

## Figures and Tables

**Table 1:** Association of endocrine-disrupting chemicals—bisphenols, parabens and triclosan—with polycystic ovary syndrome in women. Studies included in this systematic review (arranged alphabetically by first author’s last name). Statistically significant differences are presented.

Source	Study Design	Sample of Participants (No. of Women)	Sample Type	Exposure	Main Findings
Akgül et al. (2019), Turkey [84]	Case-control study	95 aged 12–18 years: 62 with PCOS, 33 controls	Blood plasma, urine samples	BPA, phthalates	- Increased urinary BPA levels in PCOS group - Positive correlation of urinary BPA levels with polycystic morphology on ultrasound
Akin et al. (2015), Turkey [85]	Cross-sectional study	173 aged 13–19 years: 112 with PCOS, 61 controls	Blood serum	BPA	- Increased serum BPA levels in PCOS group - Positive correlation of serum BPA with total T and DHEAS in PCOS group
Gu et al. (2019) [86]	Case-control study	123: 40 with PCOS, 83 controls	Urine sample	Organic UV filters, BPA, triclosan	- No association between urinary BPA and TCS and PCOS
Hossein Rashidi et al. (2017), Iran [87]	Case-control study	102: 51 with PCOS, 51 controls	First morning urine sample	BPA	- Higher urinary BPA levels in PCOS group - BPA was significantly associated with PCOS
Jędrzejuk et al. (2019), Poland [88]	Pilot study	63: 27 with PCOS, 36 controls	Blood sample	BPA	- Negative correlation between blood BPA levels and VDBP and SHBG levels in PCOS group - Positive correlation of blood BPA with bilirubin levels in PCOS and study group
Kandaraki et al. (2011), United Kingdom, Greece [89]	Cross-sectional study	171: 71 with PCOS, 100 controls	Blood serum	BPA	- Higher serum BPA levels in PCOS group - Positive correlation of serum BPA with T and A in the total group - Positive correlation of BPA with PCOS in women - Positive correlation of serum BPA with insulin resistance in PCOS group
Konieczna et al. (2018), Poland [90]	Cross-sectional study	186: 106 with PCOS, 80 controls	Blood serum	BPA	- Significantly higher BPA serum levels in PCOS group - Positive correlation of BPA levels with serum total T and FAI in PCOS group
Lazúrová et al. (2021), Slovakia [91]	Case-control study	118: 86 with PCOS, 32 controls	Fasting blood and morning urinary samples	BPA	- Higher urinary BPA levels in PCOS group women - High-BPA PCOS group demonstrated higher serum insulin and HOMA IR, lower serum estrone, estradiol, FSH and FAI comparing to low-BPA PCOS group - Positive correlation of urinary BPA levels with age in PCOS group - Negative correlation of urinary BPA levels with estradiol, T, free-T and FAI in PCOS group
Luo et al. (2020), China [92]	Case-control study	357: 119 with PCOS, 238 controls	Blood plasma	BPA, polybrominated diphenyl ethers, phthalates	- Higher BPA levels in PCOS group - Clearance of BPA, phthalates and androgens is associated with genetic polymorphisms of UGT
Šimková et al. (2020), Czech Republic [93]	Case-control study	39: 19 with PCOS (9 normal-weight, 10 obese), 20 controls	Blood plasma	Parabens, BPA, BPS, BPF, BPAF	- Higher levels of BPA in plasma of PCOS group - Higher levels of plasma BPA, AMH, LH, LH/FSH ratio, T, free T, bioavailable T, A, 7β-OH-epiandrosterone, and cytokines (IL-6, VEGF, PDGF-bb) in normal-weight PCOS women - Positive correlation of BPA with T, SHBG, FSH and LH in healthy controls - No associations of parabens and PCOS in women
Tarantino et al. (2013), Italy [94]	Cross-sectional study	60: 40 with PCOS, 20 controls	Blood serum	BPA	- Higher serum BPA levels associated with higher grades of insulin resistance, hepatic steatosis, FAI, and markers of low-grade chronic inflammation (CRP, IL-6, spleen enlargement) in PCOS group - Spleen size and FAI were the best predictors of BPA
Vagi et al. (2014), USA [95]	Case-control study	102: 52 with PCOS, 50 controls	Blood and urinary samples	Brominated diphenyl ethers, polychlorinated biphenyls, organochlorine pesticides, perfluorinated compounds, phthalates, and BPA	- No association between blood and urinary BPA levels and PCOS in women
Vahedi et al. (2016), Iran [96]	Case-control study	124: 62 with PCOS, 62 controls	Blood serum	BPA	- Higher serum BPA, triglycerides, cholesterol levels and LH/FSH ratio in PCOS group - Lower serum TSH levels in PCOS group
Wang et al. (2017), China [97]	Case-control study	38: 21 with PCOS, 17 controls	Follicular fluid and ovarian granulosa cells	BPA	- Higher BPA levels in the follicular fluid in PCOS group of women - Decreased aromatase expression and estradiol synthesis in cultured granulosa cells after in-vitro BPA exposure in PCOS group of women
Zhou et al. (2016), China [98]	Cross-sectional study	268 infertile women diagnosed with PCOS	Urinary sample	BPA	- Negative association between urinary BPA levels and AFC

Abbreviations: A, androstenedione; AFC, antral follicle count; AMH, anti-Müllerian hormone; BPA, bisphenol A; BPAF, bispheol AF; BPF, bispheol F; BPS, bispheol S; CRP, C-reactive protein; DHEAS, dehydroepiandrosterone sulfate; FAI, free androgen index; FSH, follicle–stimulating hormone; HOMA IR, Homeostatic Model Assessment for Insulin Resistance; IL-6, interleukin 6; LH, luteinizing hormone; PCOS, polycystic ovary syndrome; PDGF-bb, platelet derived growth factor bb; SHBG, sex hormone binding globulin; UGT, uridine diphosphate-glucuronosyl transferase; VDBP, Vitamin D–binding protein; VEGF, vascular endothelial growth factor; T, testosterone; TCS, triclosan; TSH, thyroid–stimulating hormone.

## Data Availability

The data are contained within the article.
